# Use of *Lactobacillus plantarum* (strains 22F and 25F) and *Pediococcus acidilactici* (strain 72N) as replacements for antibiotic-growth promotants in pigs

**DOI:** 10.1038/s41598-021-91427-5

**Published:** 2021-06-08

**Authors:** Pawiya Pupa, Prasert Apiwatsiri, Wandee Sirichokchatchawan, Nopadon Pirarat, Tanawong Maison, Anantawat Koontanatechanon, Nuvee Prapasarakul

**Affiliations:** 1 Department of Microbiology, Faculty of Veterinary Science, Chulalongkorn University, Bangkok, 10330 Thailand; 2 College of Public Health Sciences, Chulalongkorn University (CPHS), Bangkok, Thailand; 3 Department of Pathology, Faculty of Veterinary Science, Chulalongkorn University, Bangkok, 10330 Thailand; 4 Feed Research and Innovation Center, Charoen Pokphand Foods (CPF) Public Company Limited (PLC.), Chonburi, 20220 Thailand; 5 Diagnosis and Monitoring Animal Pathogens Research Unit, Chulalongkorn University, Bangkok, 10330 Thailand

**Keywords:** Microbiology, Applied microbiology

## Abstract

The lactic acid bacteria (LAB) *Lactobacillus plantarum* (strains 22F and 25F) and *Pediococcus acidilactici* (strain 72N) have appeared promising as replacements for antibiotics in in vitro﻿ studies. Microencapsulation, especially by the spray-drying method, has been used to preserve their numbers and characteristics during storage and digestion. This study compared the efficacy of these strains and their microencapsulated form with antibiotic usage on growth performance, faecal microbial counts, and intestinal morphology in nursing-finishing pigs. A total of 240 healthy neonatal pigs were treated on days 0, 3, 6, 9, and 12 after cross-fostering. Sterile peptone water was delivered orally to the control and antibiotic groups. Spray-dried *Lactobacillus plantarum* strain 22F stored for 6-months was administered to piglets in the spraydry group. Three ml of each the three fresh strains (10^9^ CFU/mL) were orally administered to piglets in each group. All pigs received the basal diets, but these were supplemented with routine antibiotic for the antibiotic group. Pigs in all the probiotic supplemented groups exhibited a better average daily gain and feed conversion ratio than those of the controls in the nursery and grower phases. Probiotic supplementation increased viable lactobacilli and decreased enterobacterial counts. Antibiotic additives reduced both enterobacterial and lactobacilli counts. Villous height and villous height:crypt depth ratio were greater in probiotic and antibiotic supplemented pigs comparing to the controls, especially in the jejunum. The results demonstrated the feasibility of using these strains as a substitute for antibiotics and the practicality of the microencapsulation protocol for use in swine farms.

## Introduction

In many parts of the world, antibiotics are regularly used as non-nutritive feed additives. This use has significantly contributed to the development of the swine industry by reducing the incidence of clinical diseases, increasing feed utilization, and promoting live-weight gain^1,2^. Unfortunately, the continuous use of antibiotics provides selective pressure to maintain and increase the emergence and dissemination of drug-resistant commensal and pathogenic bacteria, which may be transferred to both animals and humans^3–5^. This issue has become a global concern for human health. In addition, long-term antibiotic usage may cause intestinal dysbiosis and undermine gut health in the pig^6,7^. As a result, many countries have banned and restricted the inclusion of antibiotics as routine growth promotors in swine diets^8,9^, with their use restricted to controlling certain specific diseases. Based on the trend to prohibit antibiotic use in feed for growth promotion, there is an urgent need to explore alternative replacement feed additives, such as organic acids, enzymes, herbal substances, and probiotics. Probiotics are well recognized as one of the most promising alternatives to antibiotics^10,11^. Probiotics have been defined as live microorganisms promoting beneficial health effects to the host when ingested in an adequate number^12^. In general, their modes of action are mainly based on maintenance of gut integrity, stabilization of the microbiota ecosystem, antagonism to pathogenic bacteria, immune modulation, and overall health promotion, including reduction in signs of diarrhoea and improvement in growth performance^13–16^. Lactic acid bacteria (LAB) such as *Lactobacillus* spp*.*, *Bifidobacterium* spp., *Enterococcus* spp*.* and *Pediococcus* spp*.* are most frequently used as probiotics in pig production as they are believed to have beneficial effects, including reduction in the numbers of potentially pathogenic *Enterobacteriaceae* species^17^. Previously, Thai LAB strains *Lactobacillus plantarum* strains 22F, and 25F, and *Pediococcus acidilactici* strain 72N were reported to be good probiotic candidates for use in swine farms^18–20^. Nevertheless, the incorporation of probiotics into the pig production cycle is challenging, especially because of the need for storage and stability during processing and in the delivery platform. In addition, their efficacy in enhancing performance in large-scale pig production systems requires further clarification.

It is important to ensure that all probiotic strains and ready-to-use products are stable and maintain functionality until they reach the gastrointestinal tract and undergo colonization at the desired site(s) of action^21–23^. Microencapsulation has been utilized globally to preserve the shelf-life of probiotics. In particular, the spray-drying method has been used for packaging probiotics within small microcapsules to shield the probiotic cells from damaging environments. This method can be applied and scaled up easily, so that LAB are distributed homogenously in the final product within uniformly small diameter-sized microcapsules^24–27^. Alginate, which dissolves in the intestine to release entrapped cells, recently has been used to form probiotic microcapsules^28,29^. Double coating with chitosan also has an excellent film-forming ability and may improve the survival of probiotics during storage and transit in the gut^30,31^. Despite these advances, solid evidence comparing the relative efficacy of probiotics and antibiotics in improving pig performance and microbiological parameters remains scarce.

Exposure to bacteria that can colonize the gut is essential for the initial establishment of the gut microbial community. Hence, supplementation of LAB in neonatal piglets can regulate the formation of the gut microflora and consequently benefit the health of pigs^17,32^. This study aimed to evaluate the potential of microencapsulated and stored *L. plantarum* strain 22F, and fresh *L. plantarum* strain 22F, *L. plantarum* strain 25F, and *P. acidilactici* strain 72N as supplements for pigs. Growth performance and gut health parameters measured through the production cycle were compared to those in non-supplemented pigs and in pigs receiving antibiotics.

## Results

### Performance evaluation

**Figure 1:**
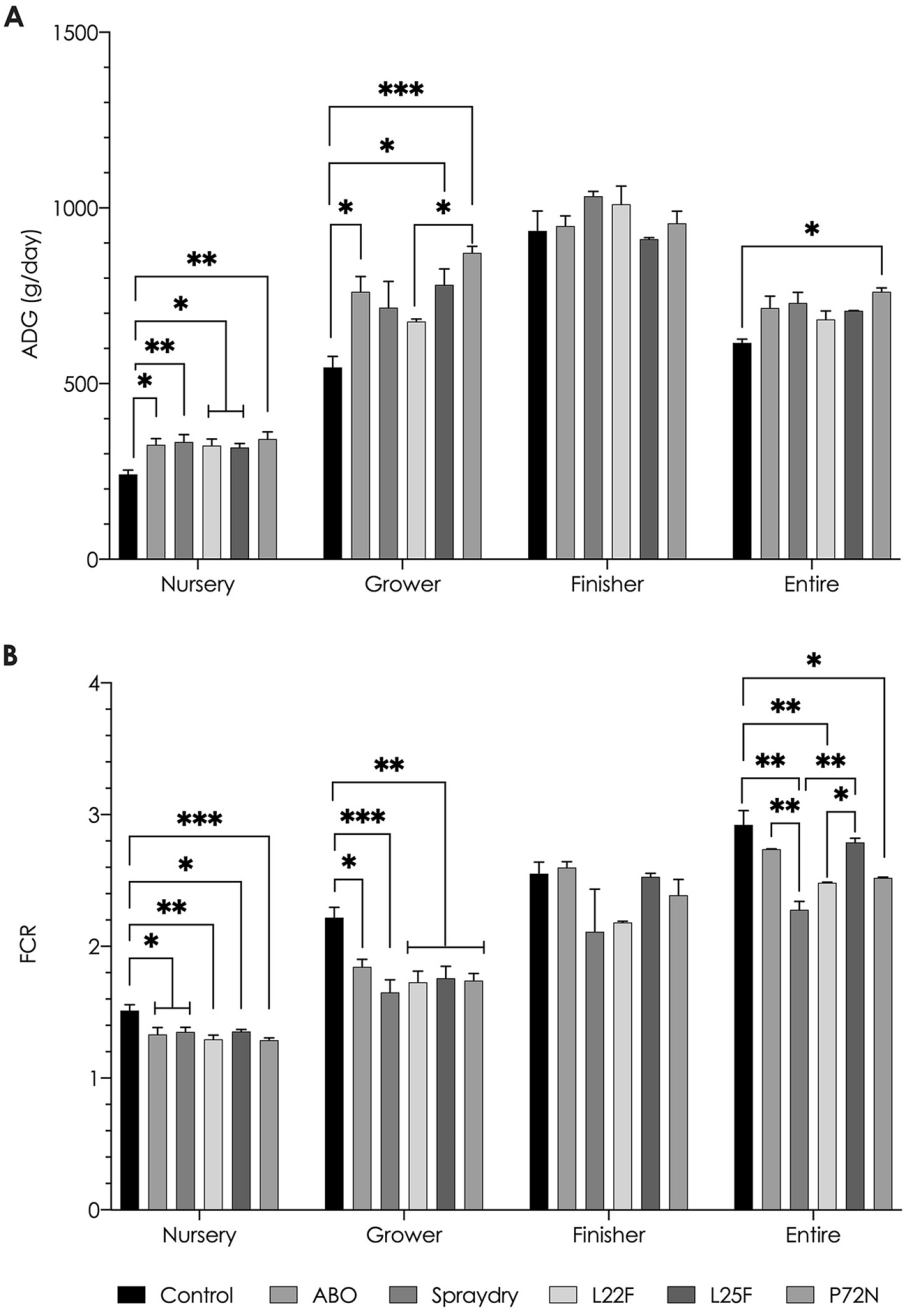
Effect of treatments on growth performance of nursery-finisher pigs. The asterisks represent statistically significant differences (*P < 0.05, **P < 0.01 and ***P < 0.001).

The initial average body weights (BW) of the pigs in kg in the six groups were 1.67 ± 0.24; 1.56 ± 0.31; 1.76 ± 0.25; 1.66 ± 0.29; 1.56 ± 0.11; and 1.74 ± 0.57, respectively, and these weights did not differ significantly. None of the pigs in this study showed clinical illness, including diarrhoea, and all survived until the end of the experiment. During the nursery and grower periods, the negative control pigs that did not received any supplementation (group1) had significantly (*P* < 0.05) lower ADG and a worse FCR than the pigs in each of the other five treatment groups, whereas no significant differences were found in the finisher phase (Fig. 1A,B). The pigs in the spraydry and P72N groups displayed the highest ADG and lowest FCR among the other experimental groups. On the other hand, when viewed over the whole experiment (nursery to finisher), only pigs in the P72N group had a significantly greater ADG than the negative control pigs (*P* < 0.05). No differences were found in either ADG or FCR amongst the five supplemented groups (including the spraydry and L22F groups, which both received *L. plantarum* strain 22F﻿); however, pigs in the spraydry group had a lower FCR over the entire experiment than the group receiving antibiotics (*P* < 0.01). The combined effect of the experiment group and the age phase impacted both ADG (*P* = 0.0004) and FCR (*P* = 0.0142) in all pigs (Supplementary Table S1).

### Return on investment (ROI) analysis

Only pigs in the P72N group showed a significantly higher BWm than those in the control group (*P* < 0.05). However, based on ROI, all the supplementations (antibiotic and probiotics) increased ROI, with P72N being the most advantageous and antibiotic supplementation being the least useful (Table 1).

**Table 1:** Body weight at market age (BWm) and ROI per pig for probiotic and antibiotic supplementation.

Experimental group	BWm (kg)	Increased^ψ^ BWm (kg)	Increased income (USD)	Total cost (USD)	Net return (USD)	ROI
Control	87.46 ± 1.04^a^	–	–	–	–	–
ABO	100.5 ± 4.26^ab^	13.04	32.73	5.00	27.73	5.55
Spraydry	102.9 ± 4.15^ab^	15.44	38.75	3.50	35.25	10.07
L22F	96.09 ± 3.09^ab^	8.63	21.66	2.00	19.66	9.83
L25F	99.37 ± 0.21^ab^	11.91	29.89	2.00	27.89	13.95
P72N	107.00 ± 1.61^b^	19.54	49.05	2.00	47.05	23.53

^abc^Means with different superscript differ significantly.

^ψ^The increased BWms were derived from the comparison of the control group with the others.

### Faecal microbial count

Pigs in the probiotic supplemented groups (spraydry, L22F, L25F, and P72N) exhibited significantly (*P* < 0.0001) higher viable faecal lactobacilli counts than pigs in the control and the ABO groups from weeks 2 to 22 (Table 2). Among the pigs receiving probiotics, those in the L25F group had the lowest number of viable lactobacilli (*P* < 0.0001). Meanwhile, viable *Enterobacteriaceae* counts were significantly (*P* < 0.0001) lower in the pigs in the probiotic supplemented and ABO groups compared to the control group. The pigs in the P72N group had the greatest reductions in viable *Enterobacteriaceae* counts (*P* < 0.0001). The pigs in the spraydry group showed less depletion of enterobacterial counts than the pigs in the L22F group (*P* < 0.0001). In all pigs, viable lactobacilli and *Enterobacteriaceae* counts diminished gradually at samplings over the 22 weeks (*P* < 0.0001).

**Table 2:** Faecal microbial profile of pigs in each experimental group.

Experimental group	Period					Mean	Significance^ψ^		
	Week 1	Week 2	Week 3	Week 8	Week 22		E	P	E*P
**Lactobacilli**									
Control	9.18 ± 0.12^ab^	7.37 ± 0.07^a^	7.29 ± 0.13^a^	6.56 ± 0.14^ab^	6.20 ± 0.05^a^	7.32 ± 0.51^A^	< 0.0001	< 0.0001	< 0.0001
ABO	9.04 ± 0.07^a^	7.57 ± 0.07^a^	7.50 ± 0.06^a^	6.84 ± 0.05^a^	6.52 ± 0.16^a^	7.50 ± 0.43^B^			
Spraydry	9.49 ± 0.09^ab^	8.51 ± 0.19^ab^	8.10 ± 0.06^bc^	7.17 ± 0.05^bc^	7.28 ± 0.03^bc^	8.11 ± 0.43^D^			
L22F	9.31 ± 0.09^ab^	8.68 ± 0.06^b^	8.00 ± 0.02^bc^	7.11 ± 0.07^ab^	7.17 ± 0.02^bc^	8.05 ± 0.43^D^			
L25F	9.27 ± 0.06^ab^	8.35 ± 0.12^b^	7.16 ± 0.06^a^	6.88 ± 0.04^ab^	7.11 ± 0.16^ab^	7.76 ± 0.46^C^			
P72N	9.60 ± 0.05^bc^	8.63 ± 0.11^b^	7.86 ± 0.12^ab^	7.19 ± 0.09^ab^	7.93 ± 0.13^bc^	8.24 ± 0.41^D^			
Mean	9.31 ± 0.08^Z^	8.19 ± 0.23^Y^	7.65 ± 0.16^X^	6.96 ± 0.10^ W^	7.04 ± 0.25^ W^				
**Enterobacteriaceae**									
Control	8.45 ± 0.02^c^	8.33 ± 0.03^c^	8.61 ± 0.10^c^	7.88 ± 0.03^c^	7.83 ± 0.09^e^	8.22 ± 0.16^D^	< 0.0001	< 0.0001	< 0.0001
ABO	8.00 ± 0.05^b^	7.55 ± 0.03^a^	7.50 ± 0.12^ab^	6.78 ± 0.11^ab^	6.55 ± 0.01^c^	7.27 ± 0.27^BC^			
Spraydry	7.94 ± 0.04^b^	7.84 ± 0.04^b^	7.59 ± 0.15^a^	6.75 ± 0.12^ab^	6.85 ± 0.10^bcd^	7.40 ± 0.25^C^			
L22F	7.75 ± 0.04^ab^	7.73 ± 0.05^ab^	7.58 ± 0.02^ab^	6.40 ± 0.04^a^	6.35 ± 0.02^ab^	7.16 ± 0.32^B^			
L25F	7.88 ± 0.03^b^	7.81 ± 0.11^abc^	7.64 ± 0.03^b^	6.84 ± 0.01^b^	6.84 ± 0.01^d^	7.40 ± 0.23^C^			
P72N	7.55 ± 0.03^a^	7.52 ± 0.10^ab^	7.21 ± 0.05^a^	6.59 ± 0.03^a^	6.22 ± 0.05^a^	7.02 ± 0.26^A^			
Mean	7.93 ± 0.12^Z^	7.80 ± 0.12^Y^	7.69 ± 0.19^Y^	6.87 ± 0.21^X^	6.77 ± 0.24^X^				

^abc^Means with different superscript differ significantly. ^ABCD/WXYZ^Means with different superscript within a column (ABCD) or row (WXYZ) differ significantly.

^ψ^Significant effects of experimental group (E), period (P) or their interaction (E*P).

### Histological analysis

**Figure 2:**
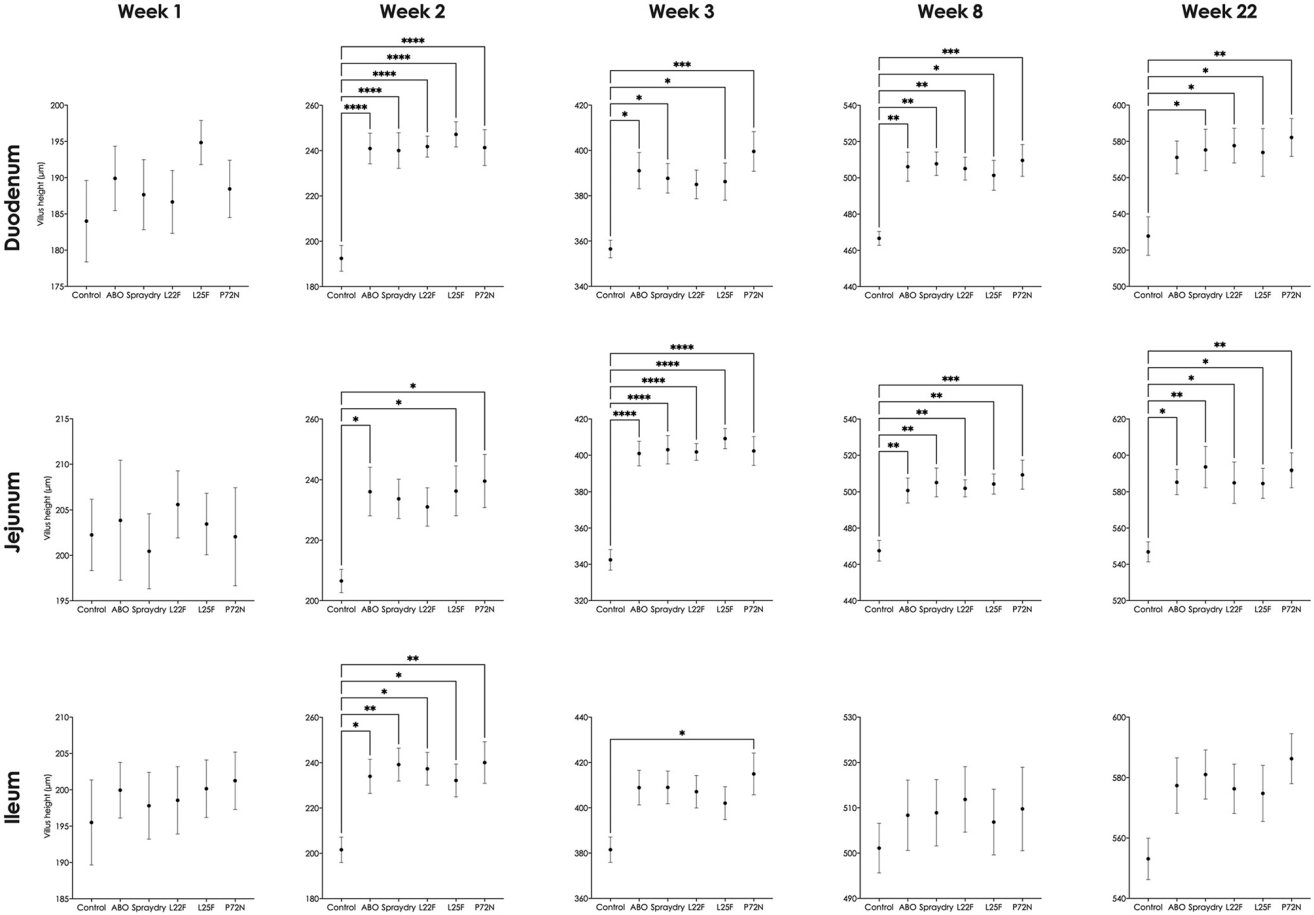
The villus height (duodenum, jejunum and ileum) of pigs in each group over the experimental period. The asterisks represent statistically significant differences (*P < 0.05, **P < 0.01, ***P < 0.001 and ****P < 0.0001).

**Figure 3:**
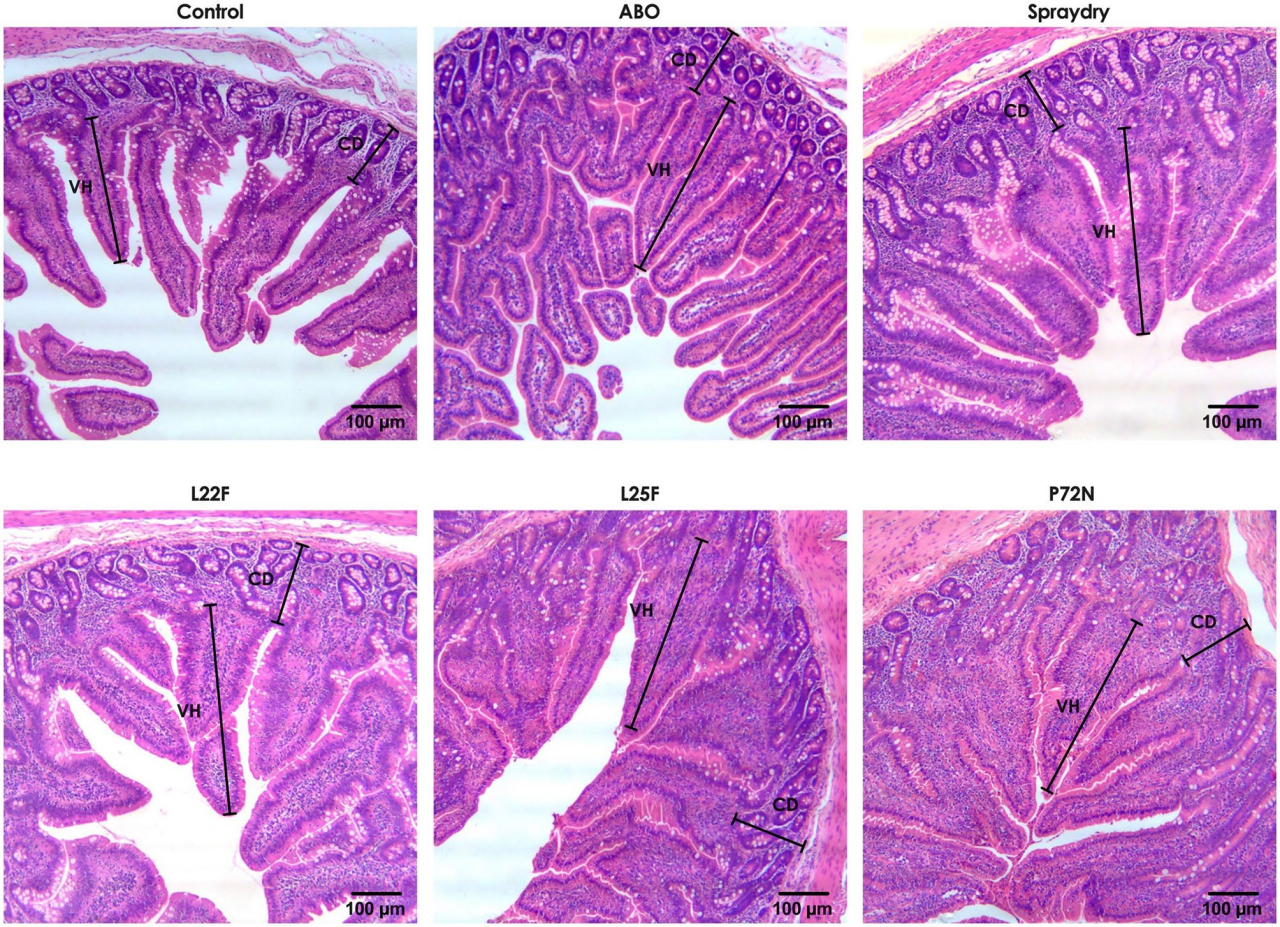
Representative intestinal morphology (jejunum) of pigs in each experimental group at week 22.

Pigs in the probiotic supplemented groups (spraydry, L22F, L25F, and P72N) had a significantly greater VH in the duodenum and jejunum than the control group over weeks 2 to 22 (Fig. 2), whereas in the ileum a greater VH was found at weeks 2 and 3 (Fig. 2). Pigs in the probiotic fed groups exhibited a significantly (*P* < 0.05) greater VH:CD ratio in the duodenum, jejunum, and ileum when comparing to control pigs (Supplementary Fig. S1). No remarkable differences were found in CD, except that pigs in the control group had a greater CD in the jejunum than the other experiment groups at week 22 (Supplementary Fig. S2). Histological examination of intestinal samples revealed some differences among the experimental groups from week 2. Pigs in the probiotic supplemented groups had similar histometric findings to the pigs in the ABO group, with all having a greater number of villi than the pigs in the control group (Fig. 3). Among the pigs receiving probiotic supplements, P72N had the best small intestinal architecture.

## Discussion

In this study, the improvements in ADG and FCR during the nursery and grower phases in healthy pigs by administering *L. plantarum* strains 22F and 25F and *P. acidilactici* strain 72N in the neonatal phase demonstrates their positive effects on growth performance and also shows that these strains are safe and suitable for swine probiotic usage. In particular, *L. plantarum* strain 22F and *P. acidilactici* strain 72N were highly effective in improving growth performance parameters. The exact mechanisms for these beneficial outcomes are likely to be specific for each probiotic strain^21,33^, and hence a combination of these probiotic strains could be even more suitable for future probiotic product development.

Comparison of our results with those of others is complicated by the fact that different studies have used different bacterial strains and pigs of different ages, housing, and health status. Our nursery phase results agree with those of Liu et al.^34^, who reported achieving a higher ADG by oral supplementation with *L. casei* 1.570, *E. faecalis* 1.2024, or a combination of the two in nursery pigs. Similarly, Yu et al.^35^ observed increased ADG by feeding *L. fermentum*, whereas Taras et al.^36^ and Ross et al.^37^ found that *Enterococcus faecium* NCIMB 10415 and a mixture of *L. amylovorus* and *E. faecium*, respectively, reduced FCR compared to untreated control pigs. On the other hand, the mixture of *L. amylovorus* and *E. faecium* given by gavage did not improve the pigs’ body weight^37^. Likewise, multispecies lactobacillus supplementation in the diet did not affect pigs’ growth performance^38^. One of our interesting findings was that the three probiotic strains administered to neonatal pigs considerably enhanced weight gain and feed efficiency into the grower phase, but not in the finisher phase. These findings were in accord with those of some other authors in relation to their use of: supplementation with *Bacillus subtilis* H4, *Saccharomyces boulardi* Sb, and LAB complex (*E. faecium* 6H2, *L. acidophilus* C3, *P. pentosaceus* D7, and *L. fermentum* NC1)^39^; addition of *B. licheniformis* (DSM 5749) and *B. subtilis* (DSM 5750) spores^40^; and supplementation with *L. acidophilus* NCDC-15 and *P. acidilactici* strain FT28^41^. In the current experiment, however, the parameters in the finisher phase of the probiotic fed groups were better than for the control group. A significantly improved ADG in pigs receiving probiotics, including spraydry, *L. plantarum* strain 22F and 25F, was not observed over the production cycle. Nevertheless, from an economic perspective, these non-significant effects could improve profitability at market age: our ROI results indicated that supplementation with spraydry, *L. plantarum* strain 22F, and strain 25F could all improve profitability, although they did not significantly improve BWm. Moreover, these probiotic fed groups had an excellent FCR over the entire experiment, highlighting their great potential for improving the pigs’ efficiency in utilizing dietary nutrients for maintenance, lean gain, and lipid accretion^42^. Previously, administration of *L. reuteri* BSA 131^43^, *E. faecium* SF68^44^, and multi-microbe probiotic (*L. acidophilus*, *B. subtilis*, *S. cerevisiae*, and *Aspergillus oryzae*)^45^ have been shown to boost pig growth performance equivalent to that achieved by administering antibiotics. Antibiotic usage might limit the effects of subclinical disease on growth, hence resulting in weight-gain benefits^17^. In the current study, no differences were observed in growth performance between pigs fed either with antibiotics or with probiotics, indicating that feeding with our probiotic strains might be a viable substitute for routine prophylactic antibiotic usage in pig farms. Importantly, the ABO pigs received one or a combination of two antibiotics over the entire production period, whereas the probiotics were only administered on five occasions in the neonatal phase. No disease was encountered in any of the pigs, including the untreated control pigs, and it is uncertain whether similar improved growth would be achieved in the face of disease challenges. Similarly, other antibiotics and different dose rates might achieve different effects on performance. Certainly, before withdrawing antibiotics from routine use, it will be necessary to assess the disease status, hygiene, and biosecurity on farms, and to carefully manage the situation accordingly.

Improvement of growth performance by using probiotics may involve several mechanisms, including stimulation of the intestinal immune system, maintaining intestinal microbiota homeostasis, and/or remedying gut health leading to digestion enhancement and improved nutrient utilization^13–15,46,47^. Interestingly, this study showed that supplementation with spray-dried *L. plantarum* strain 22F stored for 6 months increased growth performance similar to that achieved using fresh live cultures as a supplement. Wang et al.^48^ reported that supplementation with microencapsulated *L. plantarum* mixed fructooligosaccharide increased ADG in weaner pigs. Our results indicated that microencapsulation by the spray-drying method could preserve the amount and prolong the life span of the probiotic cells without impacting on their positive effect on the pig’s performance. The preservation is likely to reflect a protective effect of alginate and chitosan polymers after the microencapsulation process by forming a capsule surrounding the probiotic cells to shield them from detrimental environments^24,49^. Therefore, the microencapsulation protocol used in this study could be a prototype for up-scaling into further industrial probiotic production and practical use for livestock farms.

In this study, for practical reasons, microbial counts were made from the faeces, and it is recognized that these may not fully reflect the situation in other parts of the gastrointestinal tract where the treatments may have had effects. Nevertheless, increased lactobacilli and decreased enterobacterial cell counts in the faeces of the pigs that were fed probiotics were demonstrated in this study. It was presumed but not confirmed that the recovered lactobacilli included the strain that was administered. These results emphasize the positive effects of supplementing probiotics in enhancing beneficial components of the gut microbiota and reducing potentially harmful gut bacteria. Of the three LAB strains, *P. acidilactici* strain 72N proved best in modulating lactobacilli and *Enterobacteriaceae* numbers. LAB can make the gastrointestinal tract (GIT) healthier by maintaining the normal gut microbiota and reducing pathogens, resulting in improved health status in pigs and improving growth performance^15,46^. These outcomes are in agreement with those using *L. acidophilus* NCDC-15 and with *P. acidilactici* strain FT28^41^, and with a combination of *Bacillus subtilis* H4, *Saccharomyces boulardi* Sb, and LAB complex (*E. faecium* 6H2, *L. acidophilus* C3, *P. pentosaceus* D7, and *L. fermentum* NC1)^39^ mixed in the grower to finisher pig diet, which resulted in increased LAB and decreased *E. coli* counts. Similarly, in nursery pigs, the inclusion of *L. amylovorus* or *E. faecium* in feed resulted in a decline in enterobacterial counts^37^. In contrast, Dlamini et al.^50^ found that a mixture of *L. reuteri* ZJ625, *L. reuteri* VB4, *L. salivarius* ZJ614, and *S. salivarius* NBRC13956 incorporated in the feed pellet did not affect either LAB or other enteric bacterial counts in weaned pigs. Where there is an improvement in the proportions of the gut microbial population, this may involve several mechanisms. Firstly, probiotics modulate the microbiota’s metabolism by competition for nutritional substrates with harmful microbes. Secondly, probiotics alter the gut environment, creating acidic conditions which are less suitable for many pathogenic microbes. Lastly, they produce essential substrates that enhance the establishment of beneficial microbes^51^. Wang et al.^48^ reported that microencapsulated *L. plantarum* mixed fructooligosaccharide could modulate faecal microbial counts in pigs, as did our microencapsulated *L. plantarum* strain 22F, which was still active after 6 months. In addition, a gradual reduction in lactobacilli counts over the experiment was detected. These results coincided with the reduced growth performance in the finisher phase. Therefore, repeating probiotic supplementation at the beginning of the finisher phase may be worth considering to maintain performance. Our results showed that antibiotic usage diminished enterobacterial count as much as did probiotic feeding; however, the antibiotics also reduced the number of lactobacilli. These results indicated that antibiotic usage could induce imbalance in the microbiota because its bactericidal or bacteriostatic effects may reduce both beneficial and harmful microbes^4^. Chang et al.^43^ found that a group of pigs fed antibiotics had a similar enterobacterial count but with a reduced lactobacilli count compared to pigs fed *L. reuteri* BSA 131. Similarly, a group of weaned pigs fed a multi-microbe probiotic (*L. acidophilus*, *B. subtilis*, *S. cerevisiae*, and *Aspergillus oryzae*) had a greater number of *Lactobacillus* spp. than pigs given an antibiotic additive, although counts of harmful bacteria were similar in the two groups^45^. Probiotic supplementation can enhance the species richness and diversity of the beneficial gut microbiota, including *Firmicutes* and *Prevotella*. Both of the latter are important for the degradation of carbohydrate and hemicellulose in plant-based feedstuffs^52^, and they may promote nutrient digestibility and utilization in pigs receiving probiotics, leading to the improved growth rate. On the other hand, antibiotic administration eliminates several microbiota taxa^7,52^, impairing gut integrity and the overall health status of the microbiota. Future studies on the use of our probiotic strains in pig farms would benefit from metagenomic analysis of the intestinal microbiota to elucidate shifts in taxonomic profiles and to permit functional analysis of the microbiota.

Long intestinal villi indicate a slow enterocyte turnover and the presence of mature functional enterocytes towards the villus tips, whereas increased crypt depth and shortened villi suggest a more rapid enterocyte turnover and a less mature and functional epithelium^53^. Probiotic and antibiotic supplementation resulted in an improved VH and a greater VH:CD ratio than in control animals, suggesting improved small intestinal functionality in all treated groups. Of the treatments, *P. acidilactici* strain 72N showed the most potential to improve intestinal structure. Normally, pigs exhibit villus atrophy following the change in diet at weaning^53,54^. Probiotics improve intestinal architecture by encouraging gut maturation and lengthening of villi^55^, and this may result in an increased digestive and absorptive ability and lead to better growth performance. In agreement with the findings in our study, orally administer of *L. casei* combined with *E. faecalis* to nursery pigs resulted in an increased length of villi^34^. The study of Dowarah et al.^41^ reported greater VH in grower to finisher pigs after supplementing with *L. acidophilus* NCDC-15 or *P. acidilactici* strain FT28 in the feed. On the other hand, Lähteinen et al.^38^ found no effect of probiotic feeding on intestinal morphology in finisher pigs. In our study, the most remarkable improvement in VH and VH:CD was found in the jejunum, the main area for nutrient absorption, indicating that this might be the most important active site for our probiotic bacteria. The positive effect of the antibiotic additives on the gut structure might be explained by the antibiotics suppressing harmful bacteria in the gut that compete for nutrients and may cause some intestinal abnormalities^4^. This interpretation is concordant with the reduced enterobacterial counts found in the pigs receiving antibiotics in our study. Improvements in the intestinal structure were detected 2 weeks post-supplementation, which indicated that our probiotic strains need to be present for at least 2 weeks to allow them time to improve the morphology of the small intestine. This is similar to results in neonatal pigs where oral administration of *L. fermentum* I5007 resulted in a greater VH after 14 days^55^.

Numerous studies have demonstrated the efficacy of probiotics in pigs, although the age of the pigs involved has varied. Supplementation after weaning has been widely applied in previous studies. Suo et al.^56^ found that feeding with *L. plantarum* ZJ316 in the weaning period failed to alter the gut bacterial community. They believed that the pigs might have developed a stable microbiota after weaning, which may have been difficult to change by adding probiotics. Primal microbe colonizers are essential for establishing the gut microbial community^17,32,57^, and therefore probiotic feeding of neonatal piglets may be more effective at modulating the formation of the gut microbiota, with corresponding benefits to pig health. In our study, we administered the probiotics from an early age, and this enhanced the microbial community and improved gut integrity, resulting in better pig growth through the rearing cycle from the nursery to the finisher phase. Similarly, feeding neonatal piglets with *L. fermentum* I5007 encouraged intestinal development and altered the intestinal microbiota^55^. Thus, the protocol used in this study whereby the probiotics were administered to neonatal pigs may prove to be most effective for use on swine farms.

## Conclusion

All three of our probiotic strains are suitable for use in swine production, although *L. plantarum* strain 22F and *P. acidilactici* strain 72N appeared particularly promising. The microencapsulation protocol used in this study is practical for use in livestock farms and could be a prototype for further up-scaling into an industrial process. Administration of three fresh LAB strains and spray-dried LAB stored for 6 months resulted in beneficial outcomes similar to those achieved by the use of antibiotic additives. Hence, under the conditions of the current study, our probiotic strains were shown to be effective substitutes for antibiotics to improve growth performance in swine farms.

## Materials and methods

### Ethics statement

The study was conducted in the Feed Research and Innovation Center, Charoen Pokphand Foods (CPF) Public Company Limited (PLC.). The experimental protocols and methods in this study were carried out in compliance with the ARRIVE guidelines. The in vivo﻿ experimental study was approved according to the guidelines for experimental animals established by the Institute Animal Care and Use Committee of the Feed Research and Innovation Center of CPF (FRIC-ACUP-1707013). The use of the LAB strains was approved by the Institutional Biosafety Committee, Chulalongkorn University (IBC1631047).

The euthanasia procedures were performed following the guidelines for the euthanasia of animals complied with the American Veterinary Medical Association (AVMA). All pigs were humanely terminated by electrocution and exsanguination techniques. Briefly, pigs were rendered unconscious by electrical stunning with the head-only application. They were then immediately cut the major blood vessels in the neck, resulting in a rapid fall in blood pressure, leading to a lack of blood to the brain and death. All efforts were made to minimize the suffering.

### Bacterial strain used in the experiment

The three strains of LAB that were used were previously isolated from the faeces of antibiotic-free healthy pigs. These bacteria were identified as *Lactobacillus plantarum* (strains 22F and 25F) and *Pediococcus acidilactici* (strain 72N) and were characterized in vitro﻿ for their probiotic properties in relation to: resistance to acid and bile; lack of antimicrobial-resistance genes using European Food Safety Authority (EFSA) criteria; antibacterial properties against *E. coli* and *Salmonella*; and interference with porcine endemic diarrhoea virus^18–20^.

The probiotic bacteria were stored at − 80 °C in De Man, Rogosa and Sharpe (MRS) broth (Becton, Dickinson and Company, Maryland, USA) containing 20% glycerol. Bacterial strains were grown in aerobic conditions at 37 °C for 18–20 h in MRS medium. Each LAB strain was harvested by centrifugation (3000*g*, 4 °C, 10 min), washed, and resuspended in sterile normal saline separately to obtain a final concentration of 10^9^ CFU/mL^58^. Three milliliters of each LAB strain were orally delivered to each of the animals in the corresponding probiotic supplement feed groups on the designated days.

### Microencapsulation of probiotic strains

Previously it has been shown that of the three LAB strains, *L. plantarum* strain 22F gave the best in vitro﻿ performance^19^. Hence, this strain was selected to use in the microencapsulation procedure. Alginate (1% w/v) (Sigma-Aldrich, Missouri, USA) and chitosan (0.4% w/v) (Union Chemical 1986, Bangkok, Thailand) were used as inner and outer wall materials. A total of 10^9^ CFU/mL of *L. plantarum* strain 22F was added at a ratio of 1:5 (v/v) to alginate solution. The mixture was atomized through a spray dryer (Mini Spray Dryer B-290, Buchi, Flawil, Switzerland) with the inlet temperature set at 130 °C, and then the alginate powder was collected. One gram of this powder was added to 100 mL of chitosan solution before atomizing through the spray dryer under the same conditions as previously described. These double-coated powders containing *L. plantarum* strain 22F were recovered from the collecting vessel and stored at room temperature for 6 months before use^59^.

### Animals and housing

After cross-fostering, a total of 240 healthy neonatal pigs (Large White × Landrace × Duroc) were randomly distributed into six experimental groups, with 2 male and 2 female replicate pens per group (10 pigs per pen). The piglets were housed in an environmentally-controlled building using an evaporative cooling system. For the nursery phase, each pen (1.6 × 1.6 m) was with stainless steel floor mats and a heated plastic mat cover, a feeder, and a water nipple. For the grower and finisher phase, each pen (6 × 6 m) was with a concrete floor stall, a feeder, and three water nipples. The housing was maintained at 27 to 28 °C and 80% humidity. The photoperiod was controlled to provide 12 h of light and 12 h of dark.

### Experimental design and sample collection

The six groups of pigs comprised: Group 1 (control)—no supplementation; Group 2 (ABO)—diet supplemented with antibiotics (Table 3); Group 3 (spraydry)—supplemented with spray-dried *L. plantarum* strain 22F; Groups 4–6—supplemented with freshly prepared *L. plantarum* strain 22F (L22F), *L. plantarum* strain 25F (L25F) and *P. acidilactici* strain 72N (P72N), respectively.

**Table 3:** Ingredient composition and dietary specification of the experimental basal diet and the antibiotic usage for the antibiotic group.

Attributes	Period		
	Nursery	Grower	Finisher
**Ingredient composition (% of dry matter)**			
Broken-milled rice	51.10	37.00	42.80
Maize	–	30.00	30.00
Wheat bran	5.00	10.00	10.00
Soybean meal	33.00	15.10	9.30
Fish meal	6.00	5.50	5.50
Soybean oil	2.50	–	–
Mono-dicalcium phosphate (MDCP)	1.80	1.80	1.80
Common salt	0.35	0.35	0.35
Mineral mixture	0.25	0.25	0.25
Total	100.00	100.00	100.00
**Dietary specification**			
Crude protein	22.50	17.00	15.00
Crude fibre	4.12	3.18	3.15
Lipid	4.46	4.23	3.39
Calcium	0.59	0.46	0.41
Phosphorus	0.30	0.23	0.20
Metabolisable energy (ME; kcal/kg)	3240.00	3140.00	3120.00
**Antibiotic usage (mg/kg in feed)**			
Chlortetracycline	300.00	–	–
Amoxycillin	–	200.00	400.00
Tiamulin fumarate	–	100.00	100.00

On the designated treatment days, pigs in groups 1 and 2 were orally administered with 3 ml of sterile peptone water (Becton, Dickinson and Company, Maryland, USA) by syringe. Pigs in group 3 received 3 mL of sterile peptone water containing 1 g of double-coated *L. plantarum* strain 22F that had been stored for 6 months. Pigs in groups 4–6 received 3 ml of suspensions (10^9^ CFU/mL) of *L. plantarum* strain 22F, *L. plantarum* strain 25F, and *P. acidilactici* strain 72N, respectively. Administrations commenced on the day of cross-fostering and were repeated five times (on days 0, 3, 6, 9, and 12 after cross-fostering). The piglets were allowed to suckle sow’s milk conventionally until weaning.

On weeks 1, 2, 3, 8, and 22, ten pigs (5 males and 5 females) in each experimental group were randomly selected for collection of faeces for microbial profile analysis. Then two pigs (1 male and 1 female) from each of these ten were randomized for euthanasia, and the small intestines were collected for histological analysis. After the weaning period, pig body weight and feed intake were recorded weekly for performance evaluation. In addition, observations of morbidity and mortality were made daily throughout the experimental period. Throughout the experiment, all of the pigs had ad libitum access to tap water and a basal diet formulated following the NRC guidelines according to the pig’s body weight (Table 3). The pigs in the antibiotic group (Group 2) received the diet supplemented with the antibiotics shown in Table 3. These antibiotics and dose rates were those used in a commercial setting to control subclinical infections and improve growth rates, and had been developed on a semi-empirical basis and used over many years.

### Performance evaluation

The performance data were divided into 3 age phases: nursery (weeks 3–8), grower (weeks 8–15), and finisher (weeks 15–22). The body weight and feed intake from each experimental group were used to calculate average daily gain (ADG) and feed conversion rate (FCR). The pigs were examined daily for signs of ill-health. Moreover, the presence of any sick or dead pigs was intended to be included into the percentage of morbidity and mortality^35,37^.

### Return on investment (ROI) analysis

The probiotic and antibiotic usage performances of the pigs were estimated based on the increased body weight at market age (BWm) compared to the control group using the ROI as follows Eq. (1)^60^, where Net return represented the profit after excluding the total cost, and Total cost represented the total expense per pig for probiotic and antibiotic supplementation along the rearing cycle. The Net return, Increase income and Increased BWm were determined as follow Eqs. (2), (3), and (4), respectively. According to data from the Department of Economics and Trade, Thailand, the average liveweight price for the pigs at market age in January 2021 was 2.51 USD/kg. The total expense per pig for probiotic and antibiotic supplementation was based on the data from the manufacture of probiotic products in Thailand (K.M.P. BIOTECH CO., LTD.) and the swine raisers association of Thailand, respectively.

1 $${\text{ROI }} = {\text{ Net return/Total cost}}$$

2 $${\text{Net return }} = {\text{ Increased income }} - {\text{ Total cost}}$$

3 $${\text{Increased income }} = {\text{ Increased BWm }} \times {\text{ Average liveweight price for the pigs at market age}}$$

4 $${\text{Increased BWm }} = {\text{ BWm of the probiotic or antibiotic supplemented group }} - {\text{ BWm of the control group}}$$

### Faecal microbial count

On weeks 1, 2, 3, 8, and 22, faecal samples were obtained from the rectal swabs and placed into transport medium to maintain viability. These samples were kept on ice and immediately taken to the laboratory^61^. The samples from 10 pigs in each experimental group were pooled and mixed well with normal saline (1:9 w/v). The supernatants were subjected to serial dilution and plated at the appropriated dilution on MRS and MacConkey (Becton, Dickinson and Company, Maryland, USA) agar using the spread plate method for the determination of viable lactobacilli and *Enterobacteriaceae* cell counts, respectively. The plates were incubated at 37 °C for 48 h^37,41^. Microbial enumerations were determined in triplicate and calculated as colony forming units (CF) per g.

### Histological analysis

Small intestinal tissues (duodenum, jejunum, and ileum) taken from two pigs in each group on weeks 1, 2, 3, 8, and 22 were immediately fixed with 10% neutral-buffered formalin, dehydrated in alcohol, cleared in xylene, and embedded in paraffin wax. Embedded tissues were cut with a microtome to achieve thin sections (4–6 μm thick) and stained with hematoxylin and eosin. The tissues were examined under the light microscope for assessment of villous height (VH), crypt depth (CD), and VH:CD ratio^37,41^ using Motic^®^ Images Plus Version 2.0 (Motic, Texas, USA).

### Statistical analysis

Data from the experiments were analysed with Prism 9 for macOS version 9.0.2 (134). Effects were considered significant at *P* < 0.05. Results were presented as mean ± standard error of the mean (SEM). The means of ADG, FCR, and BWm from all replicate pens (2 male pens and 2 female pens per group) were determined for each group. Bacterial enumeration for the faecal microbial count, in log (CFU/g) units, were performed in triplicate. Twenty measurements of villi and crypts per sample were averaged to acquire VH and CD (μm) for each pig. Those parameters were used to calculate the VH:CD ratio. Analysis of data across groups was carried out using one-way ANOVA, and the comparison of means was tested by Tukey’s multiple range tests. Analyses of the combined effect of two variables, including experimental groups and age phases for the performance parameters, or experimental groups and periods for the faecal microbial count, were conducted with two-way ANOVA and Tukey’s multiple range tests.

## Supplementary Information

Supplementary Information.
