# Supplementary material for: Use of Lactobacillus plantarum (strains 22F and 25F) and Pediococcus acidilactici (strain 72N) as replacements for antibiotic-growth promotants in pigs
Source: Sci Rep. 2021 Jun 8;11:12028. doi: 10.1038/s41598-021-91427-5 (PMC8187408; doi:10.1038/s41598-021-91427-5)
Supplement: Supplementary file 1 — Supplementary Information. [file 41598_2021_91427_MOESM1_ESM.docx]

**Supplementary Information**

Use of *Lactobacillus plantarum* (strains 22F and 25F) and *Pediococcus acidilactici* (strain 72N) as replacements for antibiotic-growth promotants in pigs

Pawiya Pupa, Prasert Apiwatsiri, Wandee Sirichokchatchawan, Noppadon Pirarat, Tanawong Maison, Anantawat Koontanatechanon and Nuvee Prapasarakul


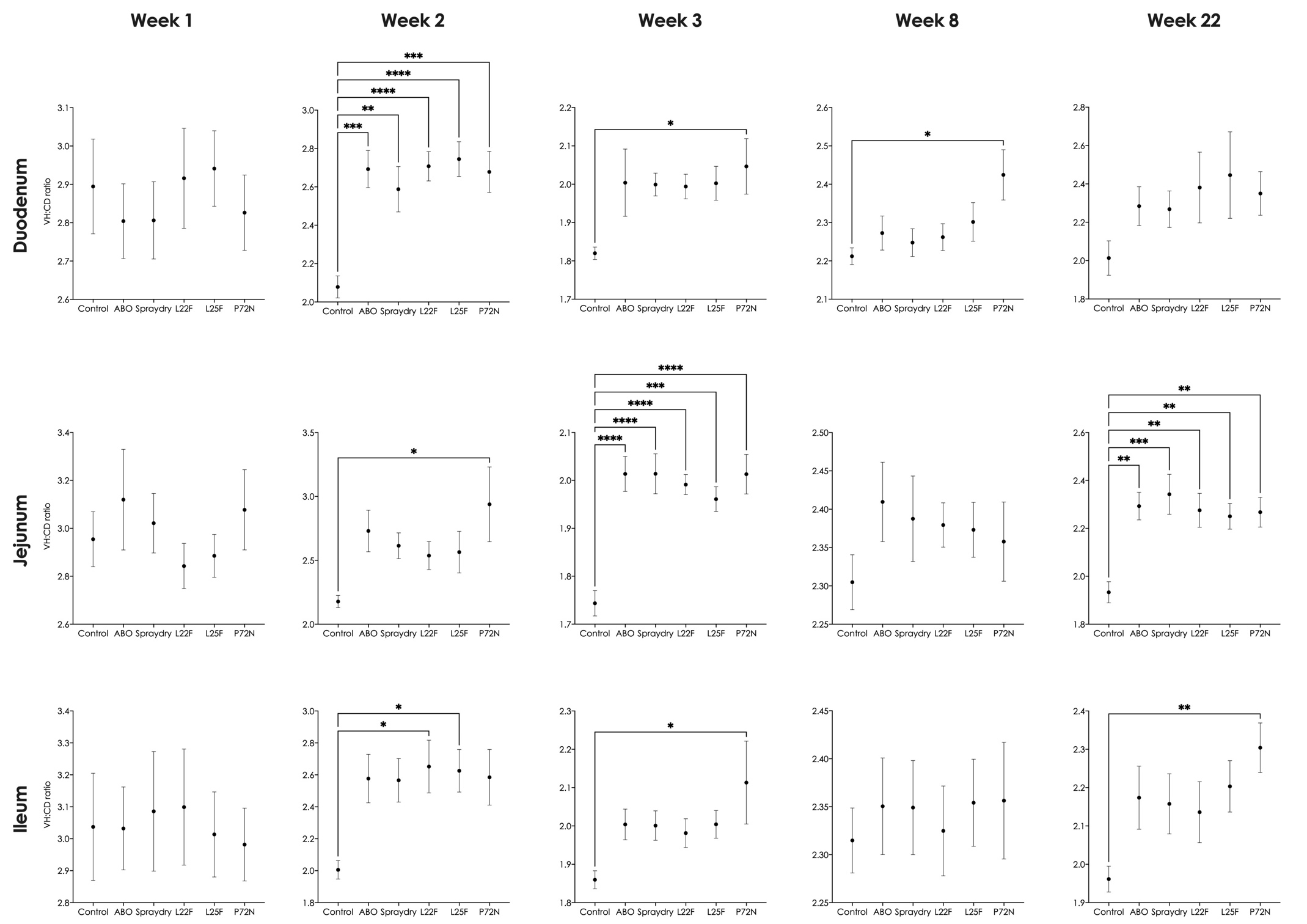


**Supplementary Figure S1.** The villus height and crypt depth ratio (duodenum, jejunum and ileum) of pigs in each group over the experimental period. The asterisks represent statistically significant differences (* = *P* < 0.05, ** = *P* < 0.01, *** = *P* < 0.001 and **** = *P* < 0.0001).


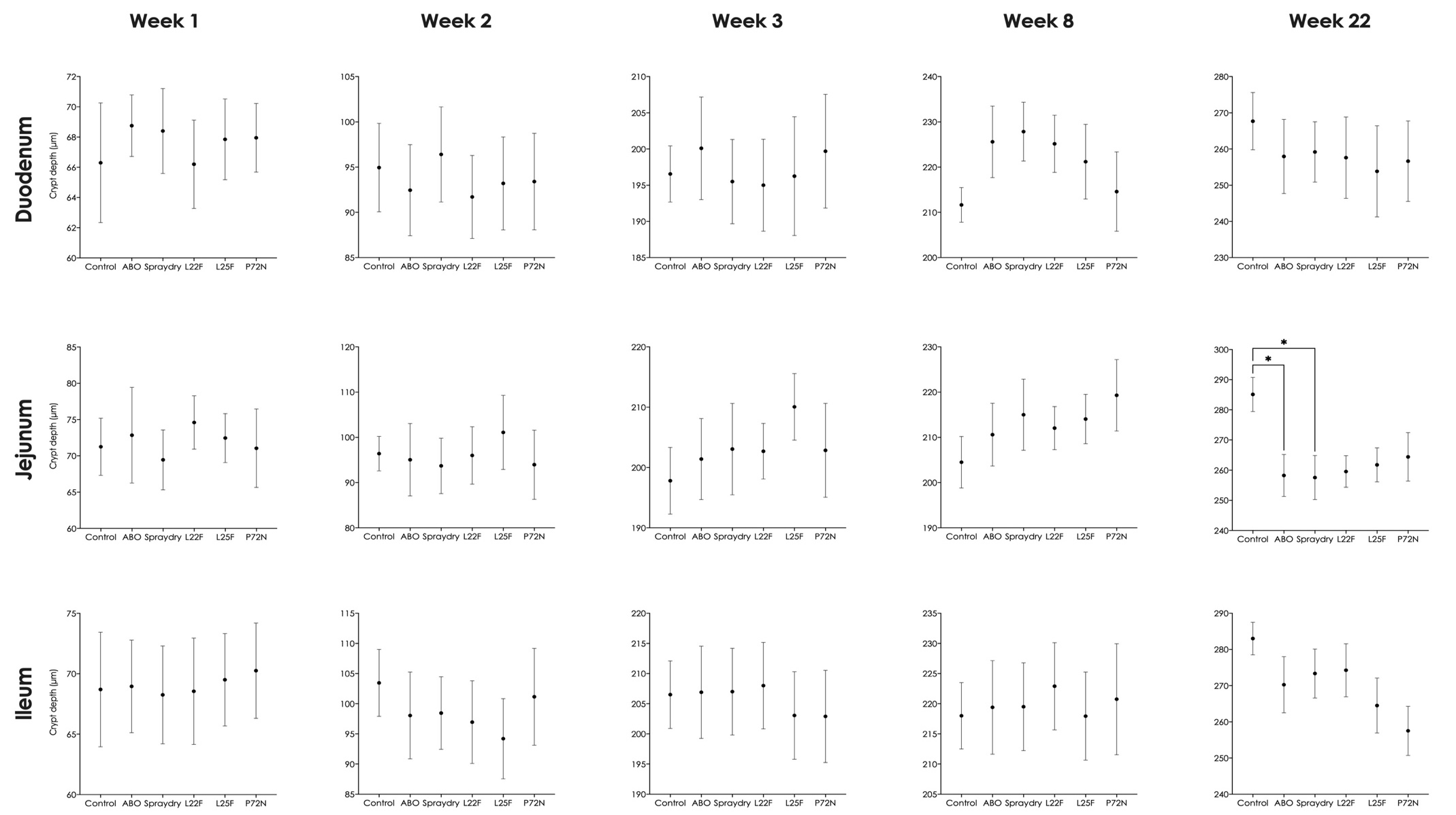


**Supplementary Figure S2.** The crypt depth (duodenum, jejunum and ileum) of pigs in each group over the experimental period. The asterisks represent statistically significant differences (* = *P* < 0.05).

| Experimental group | Age phase | | | Significance^ψ^ | | |
| --- | --- | --- | --- | --- | --- | --- |
|  | Nursery | Grower | Finisher | E | A | E*A |
| **ADG** |  |  |  |  |  |  |
| Control | 241.71±11.97^a^ | 545.72±31.63^a^ | 934.52±56.41 | 0.0002 | < 0.0001 | 0.0004 |
| ABO | 325.97±16.94^b^ | 761.04±43.53^ab^ | 948.03±28.76 |  |  |  |
| Spraydry | 333.78±20.47^b^ | 715.82±74.60^ab^ | 1032.94±13.99 |  |  |  |
| L22F | 323.48±18.43^b^ | 676.03±7.52^ab^ | 1010.23±51.48 |  |  |  |
| L25F | 317.83±11.61^b^ | 780.69±45.38^bc^ | 911.00±4.13 |  |  |  |
| P72N | 341.98±20.48^b^ | 871.68±18.64^cd^ | 955.93±34.41 |  |  |  |
| Mean | 314.13±14.89^X^ | 725.16±44.92^Y^ | 965.44±19.04^Z^ |  |  |  |
|  |  |  |  |  |  |  |
| **FCR** |  |  |  |  |  |  |
| Control | 1.51±0.05^b^ | 2.22±0.08^b^ | 2.55±0.09 | < 0.0001 | < 0.0001 | 0.0142 |
| ABO | 1.33±0.05^ab^ | 1.84±0.06^ab^ | 2.60±0.05 |  |  |  |
| Spraydry | 1.35±0.03^ab^ | 1.65±0.10^a^ | 2.11±0.32 |  |  |  |
| L22F | 1.29±0.03^a^ | 1.73±0.09^a^ | 2.18±0.01 |  |  |  |
| L25F | 1.35±0.02^ab^ | 1.76±0.09^ab^ | 2.53±0.03 |  |  |  |
| P72N | 1.29±0.02^a^ | 1.74±0.05^a^ | 2.39±0.12 |  |  |  |
| Mean | 1.36±0.13^X^ | 1.82±0.08^Y^ | 2.39±0.08^Z^ |  |  |  |

^abcd/XYZ^Means with different superscript within a column (abcd) or row (XYZ) differ significantly.

^ψ^ Significant effects of experimental group (E), age phase (A) or their interaction (E*A)

**Supplementary Table S1.** Growth performance of pigs in each experimental group during nursery to finisher phase.
